# Cross-Regional Gradient of Dendritic Morphology in Isochronically-Sourced Mouse Supragranular Pyramidal Neurons

**DOI:** 10.3389/fnana.2018.00103

**Published:** 2018-12-04

**Authors:** Zachary Logan Holley, Katherine M. Bland, Zachary O. Casey, Christopher J. Handwerk, George S. Vidal

**Affiliations:** Department of Biology, James Madison University, Harrisonburg, VA, United States

**Keywords:** dendrite, cerebral cortex, cortical layers, pyramidal neuron, layer 2/3, supragranular, arealization, *in utero* electroporation

## Abstract

Architectonic heterogeneity in neurons is thought to be important for equipping the mammalian cerebral cortex with an adaptable network that can organize the manifold totality of information it receives. To this end, the dendritic arbors of supragranular pyramidal neurons, even those of the same class, are known to vary substantially. This diversity of dendritic morphology appears to have a rostrocaudal configuration in some brain regions of various species. For example, in humans and non-human primates, neurons in more rostral visual association areas (e.g., V4) tend to have more complex dendritic arbors than those in the caudal primary visual cortex. A rostrocaudal configuration is not so clear in any region of the mouse, which is increasingly being used as a model for neurodevelopmental disorders that arise from dysfunctional cerebral cortical circuits. Therefore, in this study we investigated the complexity of dendritic arbors of neurons distributed throughout a broad area of the mouse cerebral cortex. We reduced selection bias by labeling neurons restricted to become supragranular pyramidal neurons using *in utero* electroporation. While we observed that the simple rostrocaudal position, cortical depth, or even functional region of a neuron was not directly related to its dendritic morphology, a model that instead included a caudomedial-to-rostrolateral gradient accounted for a significant amount of the observed dendritic morphological variance. In other words, rostrolateral neurons from our data set were generally more complex when compared to caudomedial neurons. Furthermore, dividing the cortex into a visual area and a non-visual area maintained the power of the relationship between caudomedial-to-rostrolateral position and dendritic complexity. Our observations therefore support the idea that dendritic morphology of mouse supragranular excitatory pyramidal neurons across much of the tangential plane of the cerebral cortex is partly shaped by a developmental gradient spanning several functional regions.

## Introduction

An attractive notion of cerebral cortical architectonics is that there is a uniform, canonical cortical microcircuitry conserved across mammalian species (Douglas and Martin, 2004). Yet, the specialization of the mammalian cerebral cortex into distinct functional areas could necessitate architectonic heterogeneity in order to adapt to distinct stimuli and information, both within individual organisms and across various species with brains adapted to different evolutionary pressures (DeFelipe et al., 2002; Elston, 2007; Herculano-Houzel et al., 2008). Systematic architectonic heterogeneity may be a particular feature of mammals, which possess supragranular cortical layers, thought to be an adaptation millions of years more recent than the infragranular layers (Jabaudon, 2017). How does architectonic heterogeneity arise, and is this pattern of heterogeneity similar across mammals? In excitatory pyramidal neurons, which account for 70%–85% of all neurons in the cerebral cortex (DeFelipe and Fariñas, 1992), anatomical heterogeneity has been observed at the dendritic arbors in many species, including primates and rodents (reviewed by Jacobs and Scheibel, 2002; Elston, 2003; Spruston, 2008; Luebke, 2017). These dendrites are physiologically important because their length can determine the number of inputs integrated per neuron, their branching patterns can determine the degree to which information is non-linearly integrated, and the size of the arbor can determine how many other neurons it receives input from (Elston, 2002). Dendrites of excitatory pyramidal neurons also possess spines that are the anatomical substrate onto which the vast majority of excitatory information is processed in the cortex (Arellano et al., 2007). Therefore, modifications to their structure can help to build diverse cortical microcircuits capable of multiple functions (Elston, 2007; Spruston, 2008; Yuste, 2011).

Dendritic morphological heterogeneity in excitatory neurons is known to arise *locally* (i.e., within a specific cortical area and layer) and *cross-regionally* (i.e., across a series of adjacent cortical areas). For example, in the rodent, an anatomically and physiologically diverse set of excitatory pyramidal neurons is found locally in supragranular layers (Larkman, 1991; van Aerde and Feldmeyer, 2013; Narayanan et al., 2017). Physiological observations also describe a heterogeneous (so-called “salt-and-pepper”) functional organization even within specific sensory regions, such as primary visual cortex (Ohki et al., 2005; reviewed in Kaschube, 2014) and primary somatosensory cortex (Sato et al., 2007). Cross-regional excitatory pyramidal heterogeneity, in which dendritic complexity varies systematically across several adjacent regions of the cortex, is a feature found in humans and non-human primates (Anderson et al., 2009; Bianchi et al., 2012; reviewed in Jacobs and Scheibel, 2002; Elston, 2003, 2007; Elston and Fujita, 2014; Luebke, 2017). For example, supragranular pyramidal neurons in visually-responsive areas of the cortex (e.g., V1, V2, V4, TEO) show a clear caudal-to-rostral increase in dendritic complexity (Elston, 2003, 2007; Elston and Fujita, 2014). This feature can equip regions within the supragranular layers with a rostrocaudal gradient of excitatory dendritic complexity across the cerebral cortex.

Like primates, mouse supragranular pyramidal morphology has been shown to differ across different functional regions (Benavides-Piccione et al., 2005; Ballesteros-Yáñez et al., 2006, 2010). Yet other results in the mouse have shown homogeneity between rostral and caudal cortical supragranular pyramidal neurons, both anatomically and physiologically (Gilman et al., 2016), particularly when compared to primates (Amatrudo et al., 2012); thus, the question of whether or not there is cross-regional heterogeneity in mice is not settled. To answer this question, we wished to use an approach that minimized selection bias, so we targeted sets of neuronal precursors highly restricted in their laminar fate to become layer II/III pyramidal neurons (Frantz and McConnell, 1996; Desai and McConnell, 2000; Shen et al., 2006) but that would be distributed across large areas of the cortical sheet. Furthermore, we used *in utero* electroporation to restrict our study to neurons born within hours of each other, as this technique preferentially targets S- and M-phase cells of the ventricular zone and subventricular zone (Stancik et al., 2010). We then tracked the morphological fate of these neurons born on the same day. Because dendritic arborization of pyramidal neurons is activity-dependent (McAllister, 2000; Cline, 2001; Wong and Ghosh, 2002; Pratt et al., 2016), and activity-dependent remodeling of dendrites is life-long (Lee et al., 2005; Chen et al., 2011), we chose to observe dendritic morphology early in life (postnatal day 23), after major arborization events are complete (postnatal day 15; Miller and Peters, 1981; Maravall et al., 2004). Our observations support the idea that dendritic morphology of mouse supragranular excitatory pyramidal neurons across much of the tangential plane of the cerebral cortex is partly shaped by a developmental gradient spanning several functional regions. Our results also strengthen the idea that mouse supragranular dendritic morphology varies along a gradient similar to that found across functional regions (e.g., visually-responsive regions like V1, V2, etc.) of several primate species (Elston, 2003, 2007; Elston and Fujita, 2014) and rodents (Elston et al., 2006).

## Materials and Methods

### Ethics Statement

This study was carried out in accordance with the principles of the Basel Declaration and recommendations of the National Institutes of Health Office of Laboratory Animal Welfare, the United States Department of Agriculture, and the Guide for the Care and Use of Laboratory Animals of the United States National Research Council. The protocol was approved by the James Madison University Institutional Animal Care and Use Committee.

### Data Sets

The reconstruction data analyzed for this study can be found as .swc files as Supplementary Material Datasheet 1 and will be uploaded and freely available on the NeuroMorpho.Org repository (RRID:SCR_002145; Ascoli et al., 2007; Akram et al., 2018). Raw confocal images that were used for semi-automated morphological analysis will be made available by the authors upon request, without undue reservation, to any qualified researcher.

### Overall Experimental Controls, Transparency and Statistical Methods

Subjective bias was minimized by acquiring images blind to morphological characteristics and exact anatomical position. The experiment included a total of 116 pyramidal neurons, taken from three independent and complete replications of the experiment (i.e., three mice, each from a different *in utero* electroporation surgery and thus from three different litters). Supplementary Table S1 describes the region and cortical depth of the neurons used for regional analysis. Cortical depth of neurons was not significantly different across the various regions (non-parametric Kruskal-Wallis test with Dunn’s multiple comparisons); an *a priori* power analysis justified acquiring a larger sample size by sampling across independent replications of the experiment (G*Power; RRID:SCR_013726). These brains were selected for their broad rostrocaudal pattern of green fluorescent protein (GFP) expression. Statistical analysis (e.g., linear regression) was performed using GraphPad Prism software (Graphpad Prism, RRID:SCR_002798). Linear regression was done by fitting a line that minimized the sum of squares of distances from the line. D’Agostino/Pearson tests were used to test for a normal distribution. When the 116 neurons were grouped into regions, the non-parametric Kruskal-Wallis test was used with Dunn’s multiple comparisons. Quantifications of grouped data are presented as Mean ± Standard Deviation. In all cases, *P* values were set with an alpha of 0.05.

### Experimental Animals, Husbandry and Housing

The animals used in this experiment were female and male C57BL6/J mice derived from a line of breeder pairs obtained from The Jackson Laboratory (IMSR cat. no. JAX:000664; RRID:IMSR_JAX:000664). After birth, the mice were housed with their parents and littermates until weaning (21 days after birth), and then with their same-sex littermates until the experimental endpoint (23 days after birth). Mice were housed in a temperature- and humidity-controlled, specific-pathogen-free environment with Teklad ${\frac{1}{4}}^{\prime \text{​}\prime }$ corncob bedding and were fed Teklad 18% protein rodent diet. The room housing the mice followed a 12-h light/dark cycle. To generate timed-pregnant mice, females that had given birth and raised a litter successfully were paired with a male the day before the peak of estrus. Mice were then separated the next day. Thus, if the female was pregnant, the day the mice were separated would be counted as embryonic day 0.5 (E0.5).

### Delivery of DNA Constructs via *in utero* Electroporation

To achieve sparse and bright labeling of supragranular neurons, the so-called “Supernova” system was used, in which a high concentration of a conditional enhanced GFP (EGFP) construct and a low concentration of a Cre recombinase construct are co-electroporated; thus, the few cells that have the Cre recombinase construct are very likely to have multiple copies of the conditional EGFP construct (Mizuno et al., 2014; Luo et al., 2017). The conditional EGFP construct (“pK038.CAG-loxP-stop-loxP-EGFP-ires-tTA-WPRE (Supernova),” Addgene cat. no. 85006; Addgene, RRID:SCR_002037) and the Cre recombinase construct (“pK031.TRE-Cre (Supernova),” AddGene cat. no. 69136; Addgene, RRID:SCR_002037) were gifts from Takuji Iwasato^1^. Constructs were amplified using standard microbiological techniques, and purified using an EndoFree Maxi kit (QIAGEN, RRID:SCR_008539). Previously described protocols were followed in order to deliver the DNA constructs via *in utero* electroporation to label a sparse population of pyramidal neurons (Vidal et al., 2016; Bland et al., 2017). In brief, a timed-pregnant mouse was deeply anesthetized at E15.5 using 1%–2.5% isoflurane in 100% oxygen. An incision was made down the midline of the abdomen in order to expose the uterus. Once the uterus had been exposed, exactly 1 μL of the DNA constructs [final concentrations of constructs: 1 mg/mL pK038.CAG-loxP-stop-loxP-EGFP-ires-tTA-WPRE (Supernova) and 10 μg/mL pK031.TRE-Cre (Supernova), in 1× phosphate-buffered saline (PBS)] was injected into a lateral ventricle of each embryo using a calibrated pipette. Following the DNA construct injection, five 50 ms electrical pulses of 50 V with a 950 ms interval were delivered to each embryo using a square wave electroporation generator (ECM 830, BTX) and 5 mm platinum-plated tweezer-type electrodes (BTX) in order to facilitate the migration of the DNA into a broad swath of ventricular and subventricular zone progenitors fated to become layer II/III pyramidal neurons (Saito, 2006; Stancik et al., 2010). The embryos and uterus were put back into their original position and the abdominal wall was sutured shut. Buprenorphine was administered intraperitoneally at 0.03 mg/kg, and the female recovered in a separate cage for several hours. After recovery, the animal was returned to its home cage, gave birth and raised its litter normally.

### Histology

Two male and one female mouse were transcardially perfused on postnatal day 23. An early age (postnatal day 23) was chosen because dendritic arbors are known to be plastic throughout life (Lee et al., 2005; Chen et al., 2011), and because major changes in dendritic arborization are complete by postnatal day 15 (Miller and Peters, 1981; Maravall et al., 2004). Each mouse was first euthanized by intraperitoneal injection of a ketamine (240 mg/kg)-xylazine (48 mg/kg)-acepromazine (1.85 mg/kg) cocktail. Once the mouse no longer exhibited any toe pinch reflexes, 20 mL of ice-cold 1× PBS followed by 25 mL ice-cold, freshly made 4% paraformaldehyde, pH 7.4 (PFA) were transcardially perfused. The skull and brain were post-fixed in 4% PFA for 24 h at 4°C. After 24 h, the solution was diluted to 1% PFA by adding 1 × PBS, and stored for up to 2 weeks at 4°C. The skull was removed from the 1% PFA solution and the brain was dissected from the skull. A coronal cut was made across the rostral end of the brain. The brain was then mounted rostral side down onto the stage of a vibrating microtome (Vibratome VT1000, Leica Microsystems). The Vibratome was then used to cut sequential 100 μm thick coronal sections. An important reason for taking coronal sections over tangential sections was to increase the amount of anatomical data acquired. *In utero* electroporation randomly targets a subset of neuronal progenitors. Consequently, it is impossible to pre-select dendritic arbors to all be confined to a specific tangential slice. Therefore, in this case, the best sectioning plane for maximum data acquisition per brain was coronal, because it was not anticipated exactly which layer II/III neurons would be labeled. Another advantage of taking coronal sections was that the apical dendritic arbors were preserved, in addition to the basal dendritic arbors. To increase the portion of the basal arbor sampled in our study, we took 100 μm sections (thicker sections would have resulted in light scatter during confocal microscopy, confounding results). The sections were mounted on glass slides with Prolong Diamond Antifade Mountant (Life Technologies) and cured for 24 h in the dark. The slides were then sealed with melted VALAP (equal parts Vaseline, lanolin and paraffin).

### Anatomical Positioning Data Acquisition and Analysis

Prepared slides were scanned for GFP at low magnification (4×) using a Nikon Eclipse Ti2 fluorescence microscope. If cells containing GFP were identified, a 4× automatically-stitched image of the entire coronal section containing the cell was then taken using the microscope and a Hamamatsu ORCA-Flash4.0 C13440 digital camera. Using FIJI software (Fiji, RRID:SCR_002285), cortical depth and distance-to-midline was determined by measuring (*x, y*) distances from the center of the soma radially out to the pial surface of the cortex (cortical depth), and from the center of the soma on the *x*-axis to the midline of the coronal section (distance-to-midline). To determine the rostrocaudal positions of the neurons, the section containing the most caudal section of the splenium of the corpus callosum was identified, and the approximate rostrocaudal distance to this section was determined by counting the number of sequential 100-μm coronal sections rostral or caudal to this section. Each coronal image was compared to the 2008 Allen Mouse Brain Reference Atlas (Lein et al., 2006,^2^) to determine the cortical region of each neuron by matching neuroanatomical landmarks and distances from the atlas to the 4× images; although shrinkage or swelling of the tissue was not observed with this histological technique, the mediolateral and dorsoventral axes of the section and the atlas were aligned and standardized to each other to account for any effects of tissue processing. Once the cortical region was determined, the distance to the closest cortical region was measured using the scale on the reference atlas. Only neurons with a high confidence of being in a cortical region (>0.25 mm distance to closest cortical region) were used for inter-regional analysis, while all other analyses were blind to putative regional identity.

### Dendritic Morphological Data Acquisition and Analysis

Using a Nikon Eclipse TE2000-E confocal microscope, Z-stack images of the full field of view were taken at 20× magnification of the complete (apical and basal) dendritic arbor from the same GFP-positive cells that were analyzed at 4× magnification. Images were taken at or above the Nyquist sampling rate in the z-direction. These Z-stack images were then used to create semi-automated 3D reconstructions of each neuron utilizing the software neuTube (Feng et al., 2015). Using the Simple Neurite Tracer plugin for FIJI (Longair et al., 2011), each path of the reconstructed neuron was assigned as being a part of the soma, the apical dendrite, or a basal dendrite. Using the assigned paths and the software L-Measure (RRID:SCR_003487; Scorcioni et al., 2008), the functions *N_stems* (number of primary dendrites), *N_bifs* (number of bifurcations in the dendritic arbor), *N_tips* (number of dendritic endings) and *length* (dendritic length) were measured for the apical and basal dendrites, respectively. The Sholl Analysis function within FIJI (Ferreira et al., 2014) was used for both apical and basal dendrites in order to determine the number of intersections at each distance from the soma. In addition to these classic parameters (Sholl, 1953), the mean value of the polynomial function used by the software to fit the Sholl analysis plot was used as a way to evaluate the average number of intersections made by the apical and basal arbors, respectively.

### Principal Axis Calculation

To account for both the rostrocaudal and the mediolateral position of a neuron as we analyzed its morphological characteristics, we plotted these positions using a model similar to Cahalane et al. (2012), but with several simplifications. First, we assigned each neuron Cartesian coordinates (*x*_1_, *y*_1_) that were its mediolateral position *x*_1_ (i.e., the distance to midline) and its rostrocaudal position *y*_1_ (i.e., the distance of the neuron’s section to the splenium of the corpus callosum, with positive numbers being more rostral). An origin (*x*_0_, *y*_0_) for the principal axis was arbitrarily assigned along the midline of the brain (*x*_0_ = 0). The relative “weight” or “importance” of the rostrocaudal position relative to the mediolateral position was arbitrarily assigned a unitless scalar *m*. The position *z* of a neuron from the origin of the principal axis was therefore given by a simple Euclidean distance formula: *z* = √(x_1_)^2^−(*m*(y_1_−y_0_))^2^). The total dendritic length was plotted against the position of all neurons on the distance from principal axis origin, and the goodness of fit to a linear model (r^2^) was measured. Both *m* and *y*_0_ were then iteratively modified until r^2^ was maximized; specific values for *m* and *y*_0_ are described in the “Results” section.

## Results

### *In utero* Electroporation Targets a Sparse Set of Supragranular Pyramidal Neurons Distributed Across the Rostrocaudal Axis of the Cerebral Cortex Without Bias to Functional Regions

To determine whether or not there is cross-regional heterogeneity in supragranular neurons of the mouse cerebral cortex, we needed to use an approach that minimized selection bias. To do so, we selected *in utero* electroporation of constructs expressing enhanced GFP because only excitatory supragranular pyramidal neurons could be labeled, meaning that all sufficiently-labeled neurons could be included in the study. This approach also had three major advantages. First, we could target a subset of neuronal precursors that would all become layer II/III pyramidal neurons. Second, knowing that this technique preferentially targets S-and M-phase cells of the ventricular zone and subventricular zone (Stancik et al., 2010), we would be able to target so-called *isochronically-sourced* neurons—that is, neurons derived from progenitors that were dividing on the same day. Third, recent developments in the technique (Vidal et al., 2016; Bland et al., 2017) and in DNA constructs specifically made with the technique in mind (Mizuno et al., 2014; Luo et al., 2017), permitted us to target sparse populations of neurons while keeping a high-contrast GFP label that would permit accurate morphological reconstructions. We therefore targeted a wide area of the developing ventricular and subventricular zone at embryonic day 15.5 and observed the dendritic morphology of neurons derived from isochronically-targeted cells on postnatal day 23, giving sufficient time for activity-dependent cues to have their major effect on the dendritic arborization of these neurons (McAllister, 2000; Cline, 2001; Wong and Ghosh, 2002; Pratt et al., 2016), which occurs by postnatal day 15 (Miller and Peters, 1981; Maravall et al., 2004).

**Figure 1:**
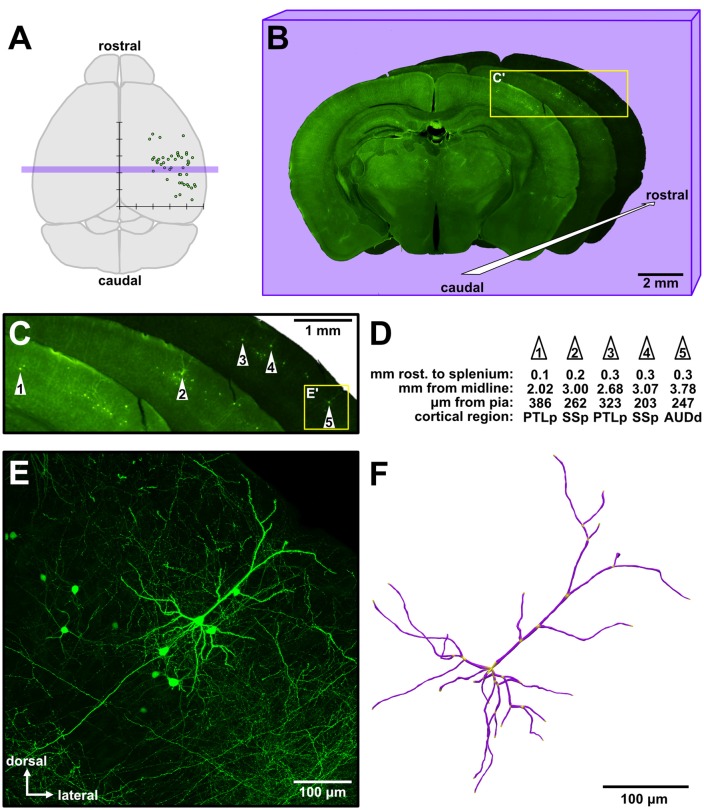
Sparse targeting of isochronically-sourced supragranular pyramidal neurons throughout the cerebral cortex permits morphological interrogation over a broad cortical area. **(A)** Schematic of the mouse brain (dorsal view). Overlaid is a graph depicting the approximate location of analyzed neurons from an experimental brain (see Figure 4A for detailed presentation of these data). The *x*-axis indicates the neurons’ distance to the midline, the y-axis indicates the relative rostrocaudal position of the neurons, and tick marks are spaced 1 mm apart. Purple rectangular overlay indicates the three coronal sections shown in **(B)**. **(B)** Example of three low-magnification images of consecutive coronal sections from an experiment, in a cascading arrangement from caudal (foreground) to rostral (background). The overall brightness of each section was altered to allow better visual distinction among the three sections. **(C)** Magnified view of the inset in **(B)**, showing the general area of green fluorescent protein (GFP) labeling in these sections. The five neurons analyzed from these sections are identified by white numbered arrowheads. **(D)** Example measurements taken from the five neurons identified in **(C)**. The first row indicates the distance of each neuron rostral to the splenium of the corpus callosum along the rostrocaudal axis. PTLp, posterior parietal association area; SSp, primary somatosensory area; AUDd, dorsal auditory area. **(E)** Medium-magnification (20×) maximum intensity Z-projection of confocal images (see Supplementary Figure S1) taken of cell #5 (inset in **C**). **(F)** A complete reconstruction of all apical and basal dendrites of the neuron in **(E)**, rendered in 3D.

**Figure 2:**
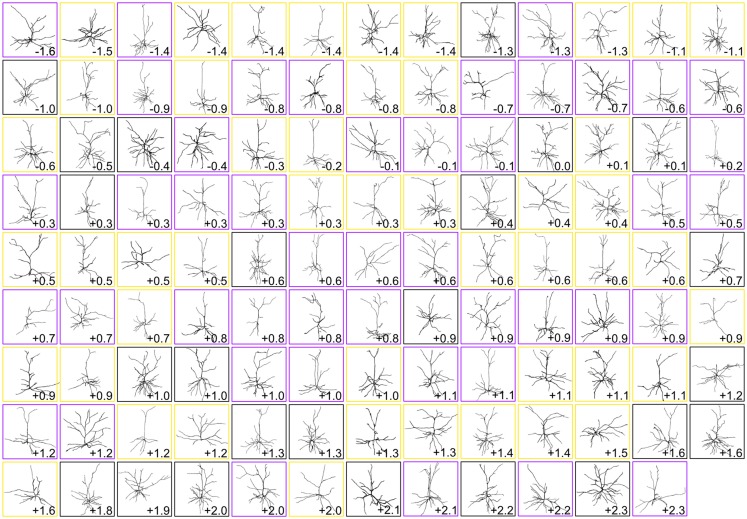
Gallery of dendritic reconstructions. All 116 pyramidal neuronal reconstructions analyzed in this study displayed with apical dendrites pointing upward. For clarity, reconstructions have been resized to fit each box and are not to scale. Neurons are arranged from most caudal (top left) to most rostral (bottom right). Neurons in boxes of the same color (black, purple, or gold) were taken from the same brain. Numbers indicate rostrocaudal distance of each neuron’s section to the splenium of the corpus callosum (negative numbers are caudal to splenium, and positive numbers are rostral to splenium).

We analyzed 116 isochronically-sourced neurons. All neurons were found only in the targeted hemispheres, were distributed throughout a large area of the cortical sheet, and were distributed across various functional cortical regions (Figure 1A). We took serial coronal sections in order to track the rostrocaudal positions of the neurons (Figure 1B), and large low-power images also permitted us to track the mediolateral position, cortical depth and putative functional region of each neuron (Figures 1C,D). Labeled neurons possessed the distinctive morphology of supragranular pyramidal neurons (Figures 1E, 2) rather than layer II upper marginal neurons (Luo et al., 2017), which was important because it is known that these structural properties of neurons are better predictors of their function than their somata’s exact location within cytoarchitectonically-defined (e.g., Nissl-stained) layers (Narayanan et al., 2017). The neurons were bright and sparse (Figure 1E, Supplementary Figure S1), permitting accurate 3D morphological reconstructions (Figure 1F). Given the fact that the mouse neocortex has approximately 5.05 million neurons per cortical hemisphere (Herculano-Houzel et al., 2013), only 1 out of approximately every 100,000 neurons was labeled.

### Supragranular Pyramidal Neuronal Morphology Appears Uniformly Heterogeneous Across the Rostrocaudal Axis, Cortical Depth and Functional Regions of the Mouse Cerebral Cortex

**Figure 3:**
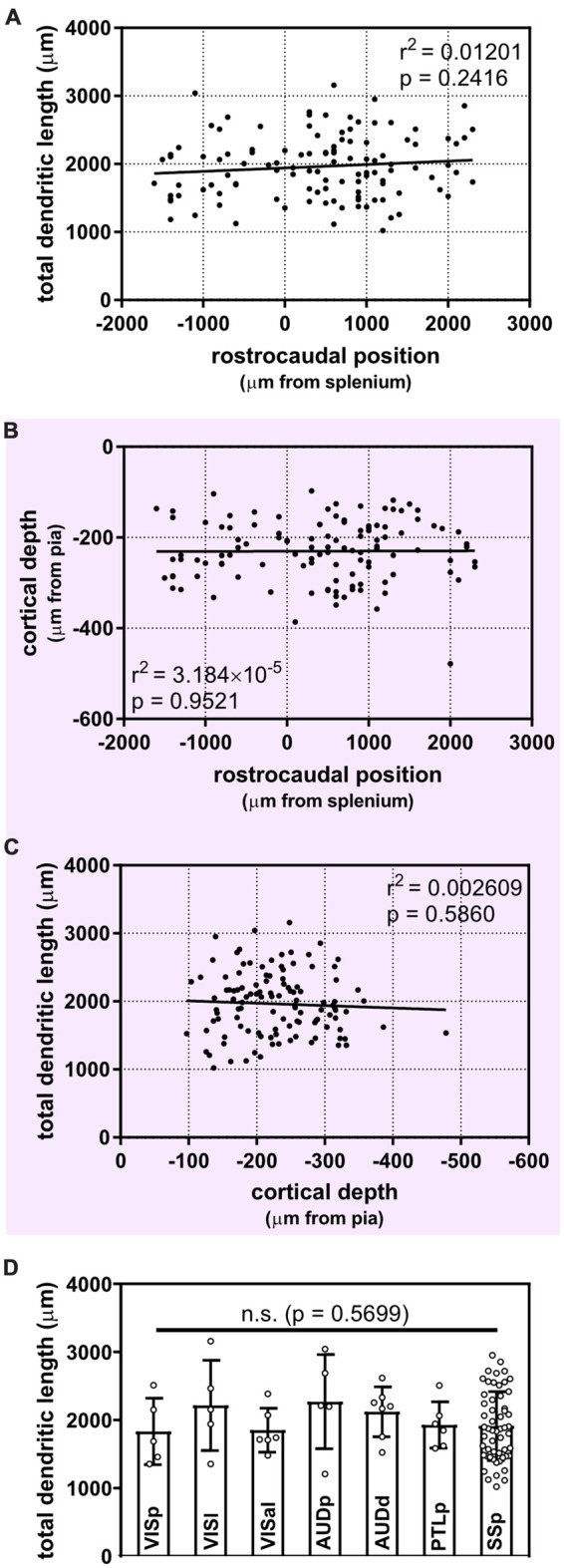
Uniform morphological heterogeneity in supragranular pyramidal neurons across the rostrocaudal axis, cortical depth and functional regions of the mouse. **(A)** No apparent relationship between the position of neurons along the rostrocaudal axis and their total dendritic length (linear regression). **(B)** Cortical depth of targeted supragranular cells does not appear to differ systematically across the rostrocaudal axis of the mouse cortex (linear regression). **(C)** No apparent relationship between depth of supragranular neuron and total dendritic length (linear regression). **(D)** No inter-regional differences observed across targeted regions (Kruskal-Wallis with Dunn’s multiple comparisons). VISp, primary visual area; (*N* = 5 neurons), VISl, lateral visual area; (*N* = 5), VISal, anterolateral visual area; (*N* = 6), AUDp, primary auditory area; (*N* = 5), AUDd, dorsal auditory area; (*N* = 7), PTLp, posterior parietal association area; (*N* = 6), SSp, primary somatosensory area; (*N* = 55). One neuron in ectorhinal cortex and 1 neuron in supplementary somatosensory area were not included in analysis. For **(A–C)**, *N* = 116 neurons.

Once we had acquired our data, our first question was whether or not the dendritic complexity of mouse supragranular neurons was correlated with their rostrocaudal position. Thus, we ordered these neurons according to their absolute position relative to an anatomical landmark of the brain (the splenium of the corpus callosum), and measured morphological features that have been shown to vary by cortical region (Ballesteros-Yáñez et al., 2006, 2010; Benavides-Piccione et al., 2005). Our results show no overall qualitative morphological differences between rostral and caudal neurons (Figure 2), nor any quantitative relationships between rostrocaudal position and features such as total dendritic length (Figure 3A), basal dendritic length, or other measurements (Table 1A).

**Table 1:** Statistical relationships between dendritic morphological features of supragranular neurons and rostrocaudal position, cortical depth and cortical region.

	(A) Morphological features vs. rostrocaudal position		(B) Morphological features vs. cortical depth		(C) Morphological features vs. cortical region	
	*r*^2^	*p*	*r*^2^	*p*	*p* (Kruskal-Wallis)	*p* (Dunn’s multiple comparisions)
Apical length (μm)	0.005981	0.4093	0.00048	0.8154	0.7018	All comparisons > 0.9999
*n* apical bifurcations	6.25 × 10^−6^	0.9788	0.006978	0.3726	0.7836	All comparisons > 0.9999
*n* apical tips	7.05 × 10^−5^	0.9287	0.008107	0.3365	0.6437	All comparisons > 0.9999
Apical Sholl mean	0.01385	0.2083	0.1087	0.0003	0.4875	All comparisons > 0.9999
Basal length (μm)	0.000139	0.9	0.006143	0.403	0.6874	All comparisons > 0.9999
*n* basal primary dendrites	0.000272	0.8605	0.02209	0.1114	0.1683	0.6199 (AUDd vs. PTLp),
						0.9638 (AUDd vs. SSp), All others > 0.9999
*n* basal bifurcations	0.000439	0.8234	0.02403	0.0966	0.5878	All comparisons > 0.9999
*n* basal tips	0.003782	0.512	0.031	0.0587	0.4414	All comparisons > 0.9999
Basal Sholl mean	0.000386	0.8341	0.01744	0.1576	0.0471	0.5678 (VISl vs. SSp),
						0.7563 (AUDd vs. SSp), All others > 0.9999
Total length (μm)	0.01201	0.2416	0.002609	0.586	0.5699	All comparisons > 0.9999

*Linear regressions used rostrocaudal position (A) or cortical depth (B) as the x-value, and the morphological parameters in each row as the y-value. Apical length is defined as the total length of all parts of the apical dendritic arbor. Apical bifurcations are the number of branch points of the apical dendritic arbor. Apical tips are the number of termination points in the entire apical dendritic arbor. Apical Sholl mean is the mean value of the polynomial fit to the Sholl profile of the apical dendritic arbor (see “Materials and Methods” section). Basal primary dendrites are the number of other dendrites coming out of the soma that are not the apical dendrite. Total length is the sum of the apical length and basal length. N are the same as Figure 3 (116 neurons)*.

It is possible that isochronically-sourced neurons have distinct laminar positioning depending on their rostrocaudal position, and that differences in laminar positioning are correlated with morphological parameters (Rojo et al., 2016), which would obscure any correlation between rostrocaudal position and dendritic complexity. However, we found no relationship between rostrocaudal position of the neurons and their cortical depth (Figure 3B), nor did we find that the cortical depth of isochronically-sourced neurons was related to their apical or basal dendritic morphology (Figure 3C, Table 1B), with the exception of the Sholl profiles of apical dendrites (Table 1B).

Finally, we found that most (89/116) neurons we analyzed were well within the confines of specific functional cortical regions, including primary and secondary visual and auditory cortex, primary somatosensory cortex, and association cortex (Figure 3D, Supplementary Table S1). As expected, many neurons were in primary somatosensory cortex, which accounts for approximately 25% of all cortical neurons (Herculano-Houzel et al., 2013). The remaining 27 neurons were associated with a specific functional region, but were within 0.25 mm of a border to another region. To determine whether or not the location of a neuron in a specific functional region was associated with their dendritic morphology, we compared the morphological features of neurons well within regional confines. “Border neurons” were not included in our analysis to account for variability in the size of cortical regions from mouse to mouse, as well as any experimental error as neurons were mapped to a reference atlas to determine the putative functional region they were associated with. Our results show no statistically significant differences in any morphological feature across any of the regions analyzed (Figure 3D, Table 1C).

### A Caudomedial-to-Rostrolateral Gradient Explains a Significant Amount of Variance in the Dendritic Morphological Features of Supragranular Pyramidal Neurons

**Figure 4:**
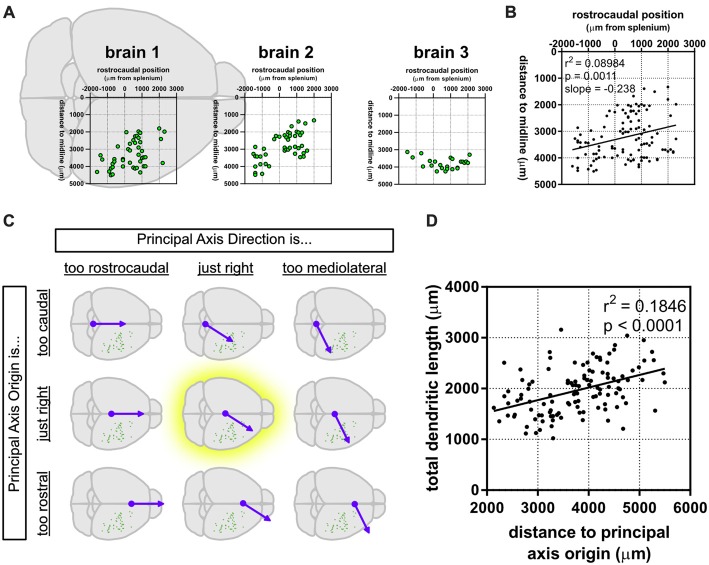
A caudomedial-to-rostrolateral gradient reveals a significant relationship between the cortical position of supragranular neurons and their dendritic complexity. **(A)** Rostrocaudal and mediolateral positions of supragranular neurons in targeted brains. “Brain 1” plot is superimposed on a schematic of a mouse brain and is the same as the data presented in Figure 1A. **(B)** Rostrocaudal and mediolateral positions of all targeted neurons are correlated. **(C)** A gradient combining both the rostrocaudal and mediolateral axes was generated and modified to maximize the correlation coefficient (r^2^; see “Materials and Methods” section). **(D)** Significant correlation between distance to the principal axis origin and total dendritic length (linear regression). *N* = 116 neurons.

We noticed that the GFP-labeled neurons were not simply randomly dispersed throughout the rostrocaudal axis of the brain, but that they had a roughly caudolateral-to-rostromedial distribution pattern (Figures 4A,B). This reminded us that developmental gradients of morphogens and transcription factors are known to have not only a rostrocaudal aspect, but also a mediolateral component to their patterning, especially during early cortical development. Thus, we asked whether the dendritic morphology of neurons might actually depend on two components: rostrocaudal position and mediolateral position. This idea is similar to how neuronal density appears to be distributed as a gradient, or “principal axis,” across the non-human primate cortex (Cahalane et al., 2012). To test this, we devised a simple gradient that accounted for both the rostrocaudal and mediolateral positions of the neurons (see “Materials and Methods” section). Results show that our data were best fitted by a caudomedial-to-rostrolateral gradient (Figure 4C), with a gradient having a principal axis axis passing through the midline 350 μm caudal to the splenium of the corpus callosum (*y*_0_ = −350 μm) and with an angle of 32.66° from the midline (*m* = 1.56). Significant relationships between the position of the neurons along this gradient and many of their dendritic morphological characteristics were revealed (Figure 4D, Table 2).

**Table 2:** Ordering neurons by distance to principal axis origin reveals significant relationships between neuronal position and basal dendritic morphological properties.

	*r*^2^	*p*
Apical length (μm)	0.000132	0.9026
*n* apical bifurcations	0.001715	0.6589
*n* apical tips	0.002147	0.6214
Apical Sholl mean	0.02015	0.1285
Basal length (μm)	0.2701	<0.0001
*n* basal primary dendrites	0.1347	<0.0001
*n* basal bifurcations	0.1884	<0.0001
*n* basal tips	0.2315	<0.0001
Basal Sholl mean	0.1455	<0.0001
Total length (μm)	0.1846	<0.0001

*Linear regressions used distance to principal axis origin as the x-value, and the morphological parameters in each row as the y-value. Definitions of morphological parameters as in Table 1. N = 116 neurons*.

**Figure 5:**
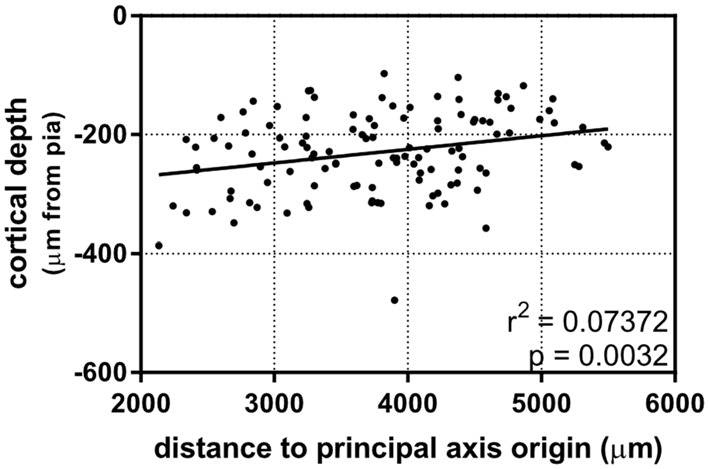
A caudomedial-to-rostrolateral gradient reveals a correlation between laminar positioning and areal position in supragranular neurons born on the same day. Rostrolateral neurons appear to be positioned more superficially when compared to more caudomedial neurons (linear regression). Principal axis and origin is the same as in Figure 4. *N* = 116 neurons.

After establishing that dendritic complexity was correlated with a caudomedial-to-rostrolateral gradient, we asked whether the cortical depth of neurons depended on this gradient, as well. Our results show a slight relationship between the two (Figure 5), suggesting that *in utero* electroporation at E15.5 targets progenitor cells that could vary systematically in their laminar fates in a caudomedial-to-rostrolateral fashion.

### Dividing the Cerebral Cortex Into a “Visual” and “Non-visual” Zone Also Accounts for Variance in Dendritic Complexity

**Figure 6:**
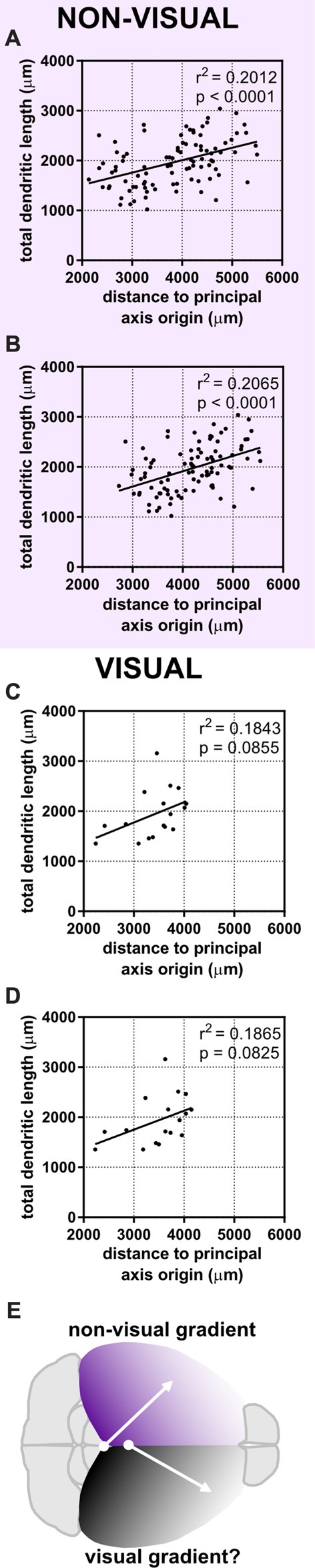
Dividing the cortex into two “zones” maintains a statistical relationship between neuronal position and dendritic complexity in the non-visual zone. Neurons were categorized as “visual” or “non-visual” (see “Materials and Methods” section). **(A)** Original gradient (used in Figure 4, Table 2) fits non-visual data better than data including both visual and non-visual neurons. **(B)** Gradient optimized for the position of non-visual neurons alone. **(C)** Original gradient (used in Figure 4, Table 2) fits visual data worse than aggregated data from all neurons. **(D)** Gradient optimized for the position of visual neurons. **(E)** Schematic of putative developmental gradients for the non-visual zone (top hemisphere) and visual zone (bottom hemisphere). For **(A,B)**, *N* = 94 non-visual neurons at least 0.25 mm from the visual/non-visual border. For **(C,D)**, *N* = 17 visual neurons at least 0.25 mm from the visual/non-visual border.

The dependence of a large amount of variance of the morphological characteristics we observed on a caudomedial-to-rostrolateral gradient reminded us of recent studies that determined neuronal density in the cerebral cortex of humans (Ribeiro et al., 2013) and mice (Herculano-Houzel et al., 2013). In both of these studies, the distribution of neuronal density in the gray matter of the cerebral cortex could not be explained by one factor alone, but rather by dividing the cortex into two “zones”: a visual/occipital zone and a non-visual/non-occipital zone. To test whether the dendritic complexity of mouse neurons was similarly organized, we categorized neurons as “visual” (i.e., neurons in primary and secondary visual areas and at least 0.25 mm from the visual/non-visual border, *N* = 17) or “non-visual” (all other neurons at least 0.25 mm from the visual/non-visual border, *N* = 94). Then, we tested their goodness of fit across the caudomedial-to-rostrolateral gradient established above. We noticed that, despite a lower sample size, the data from non-visual neurons still fit this gradient with similar statistical power (Figures 6A,C). We then modified the parameters of the gradient to fit the non-visual data better (Figure 6B, Table 3A) and found that the data were best fit with a gradient with a principal axis passing through the midline 1,650 μm caudal to the splenium of the corpus callosum and with an angle of 43.60° from the midline (Figure 6E, top hemisphere). Although data from the visual neurons did not have a statistically significant correlation to the established gradient, we nonetheless similarly modified the gradient and found that the data could be modeled better by a principal axis passing through the midline at 250 μm caudal to the splenium of the corpus callosum and with an angle of 29.74° from the midline (Figures 6D,E, bottom hemisphere; Table 3B). In both non-visual and visual areas, the direction of the gradient remained caudomedial-to-rostrolateral.

**Table 3:** Dividing the cortex into two “zones” reveals stronger relationships between neuronal position on the cortical sheet and basal dendritic morphological properties in the non-visual zone.

	(A) Non-visual zone morphological features vs. distance to principal axis origin		(B) Visual zone morphological features vs. distance to principal axis origin	
	*r*^2^	*p*	*r*^2^	*p*
Apical length (μm)	0.000466	0.8363	0.03359	0.4813
*n* apical bifurcations	0.004145	0.5376	0.01281	0.6654
*n* apical tips	0.004578	0.517	0.009245	0.7135
Apical Sholl mean	0.02703	0.1133	0.02334	0.5583
Basal length (μm)	0.2794	<0.0001	0.2197	0.0577
*n* basal primary dendrites	0.2171	<0.0001	0.001273	0.8918
*n* basal bifurcations	0.1957	<0.0001	0.122	0.1693
*n* basal tips	0.2462	<0.0001	0.1418	0.1363
Basal Sholl mean	0.1814	<0.0001	0.2088	0.0652
Total length (μm)	0.2065	<0.0001	0.1876	0.0825

*Linear regressions and morphological parameters as defined in Table 1. N = 94 non-visual and 17 visual neurons*.

**Figure 7:**
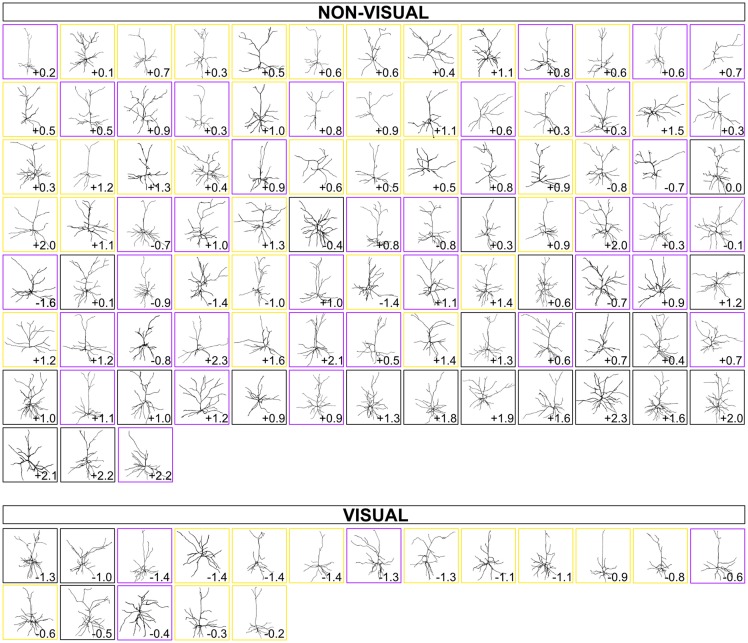
Gallery of dendritic reconstructions, ordered by distance on optimized principal axes and divided into “non-visual” and “visual” zones. For each cortical zone, neurons presented in Figure 2 are now arranged from most caudomedial (top left) to most rostrolateral (bottom right) along the principal axes defined in Figure 6 (non-visual and visual). Neurons in boxes of the same color (black, purple, or gold) were taken from the same brain. Numbers, as in Figure 2, still indicate rostrocaudal distance of each neuron’s section to the splenium of the corpus callosum (negative numbers are caudal to splenium, and positive numbers are rostral to splenium).

Our quantitative observations established a possible non-visual/visual divide and a caudomedial-to-rostrolateral patterning of dendritic complexity of supragranular neurons in the mouse cerebral cortex. We also asked whether these organizational configurations could be observed qualitatively, so we arranged a gallery of dendritic reconstructions as in Figure 2, but this time dividing them into non-visual and visual zones, and ordering them according to the gradients established in Figure 6 (Figure 7). We were surprised to notice an overall qualitative change in the complexity of the neurons’ morphology, especially when comparing the most caudomedial non-visual neurons to the most rostrolateral non-visual neurons (Figure 7).

## Discussion

In this study, we determined that a caudomedial-to-rostrolateral gradient explained a significant portion of the variance in the dendritic morphology of the supragranular neurons we observed. These findings are significant because they strengthen the idea that the mouse cerebral cortex possesses similar anatomical configurations as those in other species, particularly primates, where certain brain areas possess systematic, cross-regional dendritic morphological heterogeneity.

### A Caudomedial-to-Rostrolateral Gradient Is a Cross-Species Feature That Can Explain Variance in Dendritic Complexity, Neuronal Density and Neurogenesis in Portions of the Cerebral Cortex

Our major finding was that a simple caudomedial-to-rostrolateral gradient was sufficient to explain a significant amount of variance in several individual morphological features of the dendrites of supragranular neurons in the mouse cerebral cortex. The idea of a gradient that could shape various developmental aspects of supragranular pyramidal neurons is certainly not new. For example, several transcription factors that are involved in arealization and cortical patterning (such as Emx2, Lhx2, COUP-TFI and Bhlhb5) are largely expressed as a gradient in more superficial cortical layers (Hamasaki et al., 2004; Armentano et al., 2007; Joshi et al., 2008; O’Leary and Sahara, 2008). There is a precisely-ordered caudomedial-to-rostrolateral gradient in terms of neurogenesis in the mouse (Smart, 1983; Miyama et al., 1997; Takahashi et al., 1999; Suter et al., 2007) and the ferret (McSherry, 1984; McSherry and Smart, 1986; Jackson et al., 1989), and a similar neurogenic gradient in the cat (Luskin and Shatz, 1985). Accordingly, it is also known that, in addition to a rostrocaudal pattern in the density of neurons in the cortex of mice (Herculano-Houzel et al., 2013), non-human primates (Collins et al., 2010), and humans (Ribeiro et al., 2013), there is also a mediolateral component at play (Cahalane et al., 2012). Even transcription factors known to regulate supragranular dendritic branching (Cubelos et al., 2010) appear to be expressed as a gradient with mediolateral and rostrocaudal components (Nieto et al., 2004). It has been suggested that a lower neuronal density would be correlated with an increase in the volume of neuropil contributed by enlarged dendritic arbors (Cahalane et al., 2012). We find this prediction to be correct: here, we find that, in the portion of the mouse cerebral cortex we studied, rostrolateral neurons tend to be more complex than their caudomedial counterparts (Figures 4, 7).

A possible reason that the rostrolateral dendritic arbors we observed were generally larger and more complex is that cortical neurogenesis ends earlier rostrolaterally, thus giving more time for isochronically-sourced neurons that settle into more rostrolateral areas to develop their dendritic arbors (Cahalane et al., 2012) by the time we observed them, approximately 1 week after major dendritic arborization was complete (Miller and Peters, 1981; Maravall et al., 2004). Cortical depth has been considered a good indicator of the developmental age of pyramidal neurons ever since the landmark experiment by Angevine and Sidman (1961). Since then, studies using newer fluorescent tagging and RNAseq techniques have shown an even stronger tie between neuronal birthday and laminar positioning (Telley et al., 2016). However, we observed that caudomedial neurons have a tendency to be deeper than rostrolateral neurons in our data set (Figure 5), which could imply that caudomedial neurogenesis is delayed, and therefore isochronically-sourced neurons that settle into caudomedial areas of cortex are deeper than those that settle into rostrolateral areas. Nonetheless, the observed tendency of isochronically-sourced caudomedial neurons to be deeper is weak (Figure 5) and therefore we cannot suggest definitively whether there is another factor involved in laminar positioning apart from the developmental age of newborn neurons.

### Rostrocaudal Position, Cortical Depth, or Functional Region Alone Are Not Optimal Predictors of Mouse Supragranular Dendritic Complexity

We found that there was a surprising *lack* of anatomical heterogeneity in excitatory pyramidal neurons when only taking into account the rostrocaudal axis of the cerebral cortex, the entire depth of layer II/III, or when comparing across distinct functional cortical regions. This is in line with previous studies that have shown only modest anatomical and physiological differences between frontal and primary visual supragranular (layer III) cortical neurons in the mouse (Gilman et al., 2016; Hsu et al., 2017; Luebke, 2017). Our results extend the conclusions of these studies, first by including neurons located between the frontal and primary visual areas along the rostrocaudal axis, and also by including several additional functional cortical regions (e.g., primary somatosensory cortex). At the same time, our findings (at first glance) appear to challenge the conclusions made in other landmark anatomical studies of mouse cerebral cortical layer III neurons, such as dendritic morphological differences observed across various functional regions (Benavides-Piccione et al., 2005) that could even be described as having a roughly rostrocaudal configuration (Ballesteros-Yáñez et al., 2006, 2010).

However, various considerations show that our study does not challenge but rather complements these previous studies. For example, we and Gilman et al. (2016) took coronal sections to preserve the apical dendrites, either for the purposes of obtaining anatomical information about the complete dendritic arbor (this study) or for electrophysiological measurements (Gilman et al., 2016), while Benavides-Piccione et al. (2005) and Ballesteros-Yáñez et al. (2010) took tangential sections to preserve the basal dendritic “skirt” of layer III pyramidal neurons. Tangential sections eliminate the apical dendrite but minimize the chance of cutting away a section of the basal dendrite, thereby revealing small but significant differences in basal morphology that could otherwise go unnoticed (Elston et al., 2011; Elston and Fujita, 2014). Yet, the same group that did not find differences between mouse frontal and occpital dendritic morphology (Gilman et al., 2016) was able to find them in non-human primates using similar methodology (Medalla and Luebke, 2015). So, while there may be anatomical differences in the morphology of basal dendrites across functional cortical regions in the mouse, as well as laminar differences (Rojo et al., 2016), the differences may have been too small to be observed in this study or by Gilman et al. (2016). Furthermore, our study was focused on minimizing selection bias and tracking the fate of isochronically-labeled neuronal progenitors. Because the resulting neurons were not distributed homogeneously across cortical regions, it is more difficult to determine whether inter-regional dendritic morphological differences exist.

Furthermore, Benavides-Piccione et al. (2005) were able to observe how dendritic morphology changed across several functional regions in the mouse after reducing the dimensionality of 156 variables using principal component analysis. This powerful, unbiased approach revealed 11 variables that accounted for much of the observed morphological variance, but this also implies that *single* morphological characteristics do not change a great deal across functional regions. This contrasts with our study, in which we can quantitatively show how single morphological parameters change depending on the position of a neuron along a simple gradient (Figure 6, Table 3).

When comparing this study to others, it is important to note that here we are attempting to establish *whether or not* there exists a cross-regional gradient of supragranular dendritic morphology in the mouse cerebral cortex, rather than the *degree to which* this configuration shapes the mouse cortex. Thus, because our study was not designed to be as sensitive to basal dendritic morphological differences, the idea that there are regional or laminar differences in the basal dendrites of supragranular mouse cortex is still valid.

### Caudomedial-to-Rostrolateral Gradient of Dendritic Complexity: a Feature Scaled up in Certain Primate Brain Regions?

The range of morphological characteristics we observed (e.g., qualitatively in Figures 2, 7, and quantitatively in Supplementary Table S2) indirectly suggests that there is a non-negligible diversity of dendritic morphology that cannot be explained by the caudomedial-to-rostrolateral gradient, in line with similarly detailed observations of rodent pyramidal neurons (Larkman, 1991). Also, in our study, parameters such as dendritic length only increase by about 50% when comparing the most caudomedial neurons to the most rostrolateral neurons of our data set (Figure 4D). In contrast, the macaque prefrontal cortex has over 16 times the number of dendritic spines than in visual cortex (Elston, 2000). Humans and non-human primates also have larger-magnitude rostrocaudal changes in dendritic complexity in certain brain regions (Elston et al., 2011; Elston and Garey, 2013), as do other rodents (Elston et al., 2006; Elston and Manger, 2014). The human cortex also has similarly dramatic configurations in dendritic topography (Anderson et al., 2009) and spine density compared to other primates (Elston et al., 2001). The decrease of the slope (i.e., “strength”) of a gradient as one moves from certain primate regions to the mouse has been observed with other cortical parameters known to have developmental gradients, such as the number of neurons per unit of cortical area (Charvet et al., 2013). The lack of a particularly “strong” gradient of dendritic complexity in mice, when compared to specific regions in brains of primates (e.g., V1, V2, V4, TEO; see Elston, 2003, 2007; Elston and Fujita, 2014) and other species (Elston, 2007), is more reasonable when considering that the lack of a strong anatomical organization does not necessarily render the microcircuit less effective. For example, Reeler mice, which have a disorganized cortical lamination (and not an inverted cortical lamination; Wagener et al., 2010), are still able to form functional circuits, even in the absence of an apparent anatomical organization (Wagener et al., 2016; Prume et al., 2018). Thus, we postulate that specific functional areas of the mouse cortex are built using additional anatomical and physiological features that, together, provide the mouse with a neural configuration that can process distinct sets of information necessary for survival.

On the other hand, we were surprised to detect significant changes in single morphological parameters along a short distance—a few millimeters. Single morphological parameters are known to change much more across specific brain regions of other species with larger cortical sheets. If our observation of a caudomedial-to-rostrolateral gradient in mouse supragranular dendritic complexity is a common feature observed across various species, then could this be a feature that is “scaled up” in certain brain regions of species with larger brains? How might this occur? As a thought experiment, let us suppose there is a species possessing a simple rostrocaudal developmental gradient regulating dendritic arborization and we find a dendritic morphological difference between rostral and caudal neurons. If we increase the brain size of this species, but conserve the developmental gradient and the effect of that gradient on dendritic morphology, then we would not expect a net change in the dendritic morphology between the rostral and caudal neurons (Elston, 2007). However, if we consider that cortical development could now take longer because of the larger brain size, one could postulate that newborn cortical neurons in these brains are exposed to the developmental gradient for a longer period of time as their dendrites arborize. Thus, we would expect a larger dendritic morphological difference between rostral and caudal neurons.

## Conclusion

Even in the mouse cerebral cortex—lissencephalic, thin, small and composed of simpler neurons when compared to humans—we have found a gradient that explains some of the observed variance in supragranular pyramidal dendritic morphology. We therefore conclude that cross-regional architectonic heterogeneity exists in the mouse supragranular cortex. Furthermore, our data provide more evidence that there is not a uniform, canonical, or generalizable excitatory microcircuitry in the mouse cortex. It is fascinating that the mouse cerebral cortex appears to possess one more of the many anatomical configurations known to exist in other species, including specific brain regions of primates. Investigating each configuration and what shapes them in each species will uncover fundamental information that will enhance our approaches to treating neurodevelopmental disorders and understanding the enigmatic workings of the human brain.

## Author Contributions

This study originated from early discussions among KB, ZC, CH and GV, which arose from data acquired by all authors. GV conceived the initial experimental design, and all authors contributed to the final experimental design. GV performed *in utero* electroporation. ZH, KB, ZC and CH completed experiments and acquired data. ZH and ZC organized the database. ZH, KB, ZC and GV analyzed the data. ZH and KB acquired most images and analyzed most reconstruction data. ZH, KB and GV wrote the manuscript. GV obtained funding and managed laboratory personnel and material directly related to the manuscript. All authors revised the manuscript, read and approved the submitted version of the manuscript.

## Conflict of Interest Statement

The authors declare that the research was conducted in the absence of any commercial or financial relationships that could be construed as a potential conflict of interest.

## Footnotes

^1^http://snsupport.webcrow.jp/

^2^http://mouse.brain-map.org/static/atlas

## Supplementary Material

FIGURE S1 Medium-magnification (20x) Z-stack of confocal images taken of cell #5 (see inset in Figure 1C).
