# Supplementary material for: Cross-Regional Gradient of Dendritic Morphology in Isochronically-Sourced Mouse Supragranular Pyramidal Neurons
Source: Front Neuroanat. 2018 Dec 4;12:103. doi: 10.3389/fnana.2018.00103 (PMC6288488; doi:10.3389/fnana.2018.00103)
Supplement: Supplementary file 2 [file Table_1.DOCX]

## Supplemental Table 1. Variability in cortical depth and region of targeted mouse supragranular pyramidal neurons.

| region | N | mean cortical depth (µm) | standard deviation (µm) |
| --- | --- | --- | --- |
| VISp | 5 | -291.3 | 28.1 |
| VISl | 5 | -259.5 | 65.54 |
| VISal | 6 | -230.7 | 50.39 |
| AUDp | 5 | -200.7 | 50.47 |
| AUDd | 7 | -232.9 | 78.4 |
| PTLp | 6 | -285.3 | 73.12 |
| SSp | 55 | -225.1 | 67.3 |

A non-parametric Kruskal-Wallis test with Dunn’s multiple comparisons showed no statistically significant differences in cortical depth across these regions (alpha set at 0.05). Only neurons over 0.25 mm from the border of another region were used for regional analysis (see Material and Methods).

## Supplemental Table 2. Variability in anatomical features of mouse supragranular pyramidal neurons distributed throughout the cerebral cortex.

|  | N | Minimum | Maximum | Mean | Standard Deviation | Coefficient of variation | Normal distribution? |
| --- | --- | --- | --- | --- | --- | --- | --- |
| apical length (µm) | 116 | 197.9 | 1460 | 832.9 | 267.7 | 32.15% | Yes (P = 0.1406) |
| *n* apical bifurcations | 116 | 2 | 18 | 7.905 | 3.334 | 42.17% | No (P = 0.0230) |
| *n* apical tips | 116 | 3 | 19 | 8.871 | 3.366 | 37.95% | No (P = 0.0298) |
| apical Sholl mean | 116 | 1.36 | 5 | 2.896 | 0.8374 | 28.92% | No (P = 0.0246) |
| basal length (µm) | 116 | 420.1 | 2409 | 1129 | 376.1 | 33.30% | Yes (P = 0.3913) |
| n basal primary dendrites | 116 | 2 | 9 | 4.379 | 1.29 | 29.45% | No (P = 0.0263) |
| *n* basal bifurcations | 116 | 1 | 24 | 10.76 | 5.043 | 46.88% | Yes (P = 0.1418) |
| *n* basal tips | 116 | 4 | 28 | 15.16 | 5.586 | 36.84% | Yes (P = 0.2283) |
| basal Sholl mean | 116 | 2.46 | 13.16 | 6.437 | 2.243 | 34.85% | No (P = 0.0119) |
| total length (µm) | 116 | 1021 | 3158 | 1962 | 462.2 | 23.55% | Yes (P = 0.2097) |

Alpha value for D’Agostino and Pearson tests for normal distribution were set at 0.05.
